# Pyridinic nitrogen dominated doping on Pd/carbon catalysts for enhanced hydrogenation performance

**DOI:** 10.3389/fchem.2022.1046058

**Published:** 2022-11-02

**Authors:** Limin He, Yangdong Wang, Can Wang, Zhicheng Liu, Zaiku Xie

**Affiliations:** ^1^ State Key Laboratory of Green Chemical Engineering and Industrial Catalysis, Shanghai Research Institute of Petrochemical Technology, SINOPEC Corp, Shanghai, China; ^2^ China Petrochemical Corporation (SINOPEC Group), Beijing, China

**Keywords:** hydrogenation, pallidium, carbon materials, pyridinic nitrogen, synergistic effect

## Abstract

The hydrogenation of 4-carboxylbenzaldehyde over Pd catalysts is a crucial process during the production of pure terephthalic acid. Herein, ZIF-8 derived carbon materials (NC) with adjustable N types were synthesized and used as the supports of Pd catalysts. Pd supported on NC with 50.5% of pyridinic N exhibited best hydrogenation activity with a TOF value of 4.1 min^−1^. The microstructures of NC support and electronic structures of Pd in Pd/NC were investigated by techniques such as XRD, N_2_ physisorption, XPS, H_2_-O_2_ titration and TEM. The nitrogen species in CN surface not only can adjust chemical state and dispersion of Pd nanoparticles (NPs), but also promote the adsorption and activation capability of H_2_ molecular. Besides, the ratio of Pd^0^/Pd^2+^ and Pd dispersion were closely correlated with pyridinic nitrogen content. The improvement in hydrogenation activity and stability of Pd/CN catalyst in relative to Pd/C were ascribed to the synergistic effect of pyridinic nitrogen and active site Pd^0^.

## Introduction

Terephthalic acid (TA) as an important intermediate was widely used for the manufacture of polyethylene terephthalate. Industrially, 4-carboxy benzaldehyde (4-CAB) was the main impurities of crude terephthalic acid. The remove of 4-CBA was conducted by the catalytic hydrogenation of 4-CBA to obtain p-toluic acid over Pd catalysts. (Jhung et al., 2002). So far, activated carbon supported Pd catalysts remain the most efficient catalyst in actual industrial applications (Menegazzo et al., 2007; Zhu et al., 2015). Nevertheless, the stability of Pd/C is not satisfactory and the aggregation of Pd NPs is severe due to the weak interaction of Pd and carbon. As is well known, the nature of the supports played a strong influence on the structural properties of Pd catalysts and their catalytic hydrogenation performance. So far, various materials such as SiO_2_ (Li et al., 2008), SiC (Zhou et al., 2012; Tie et al., 2018), TiO_2_ (Kan et al., 2012; Liu et al., 2018) and carbon materials (Wang et al., 2015; Li et al., 2018) were applied as the supports of Pd for the catalytic hydrogenation of 4-CBA. Among various supports, carbon materials were still ideal supports of Pd due to their good corrosion resistance, excellent thermal stability and good chemical inertness (Wang et al., 2022; Zhou et al., 2022). Recently, nitrogen doped material demonstrated fantastic catalytic performance in various fields (Siuzdak et al., 2015; Wang et al., 2017; Wang et al., 2019; Lobiak et al., 2020; Shi et al., 2020), especially in the heterogeneous catalytic hydrogenations or oxidations (Xu et al., 2012; Lu et al., 2019; Hao et al., 2021; Wang et al., 2021). The incorporation of nitrogen atoms into carbon materials or metal oxide had a significant impact on the chemical and electronic properties of metal catalysts, such as dispersion and electronic densities of metal nanopaticle, chemical state, active sites, etc (Cui et al., 2016; Shen et al., 2020; Mao et al., 2019; von Deak et al., 2012; Wood et al., 2014). In early study, Wilson et al. reported the introduction of amine groups in stone-fruit activated carbon can increase the proportion of Pd^0^, resistant to re-oxidation (Radkevich et al., 2008). In contrast, Xia et al. revealed that pyridinic or pyrrolic nitrogen atoms showed strong interact with Pd, leading to the high proportion of Pd^δ+^ species over nitrogen doped Pd catalysts (Jiang et al., 2016)). Bulusheva et al. synthesized Pd catalysts supported on nitrogen functionalized mesoporous carbon, and confirmed that the surface concentration of Pd^0^ decreased after N doping (Bulushev et al., 2016). Besides, nitrogen doped activated carbon synthesized with dicyandiamide as nitrogen source and as supports of Pd catalysts (Nie et al., 2016), the percentage of Pd^0^ decreased obviously from 78.6% to 15.5% and then increased to 27.7%. Generally, nitrogen dopant in carbon materials mainly existed four types. They were pyridinic N, pyrrolic N, graphitic N, and N-oxide respectively (Yang et al., 2014; Cao et al., 2017; Podyacheva et al., 2017). Because of the complicacy of different types of N doping, the effects of nitrogen doping on the electronic states of metal catalysts have remained elusive and no consensus.

In our previous work (He et al., 2021), we introduced nitrogen atoms into activated carbon to stabilize Pd NPs and adjust their chemical and electronic structure using urea as nitrogen resource. The ZIF-8 framework, which has uniform ordered pore structure and rich nitrogen element, was an ideal nitrogen resource to prepare nitrogen doped materials by calcination under an N_2_ atmosphere. Inspired by these results, AC@ZIF-8 hybrid nanocomposites were prepared by the growth of ZIF nanocrystals on the surface of AC. And then, a series of nitrogen doped carbon materials (NC) with adjustable N types and N contents have been synthesized by the carbonization of ZIF-8 based carbon materials. Pd on different NC samples were also synthesized and tested for the hydrogenation of 4-CBA under mild reaction conditions. The effects of nitrogen doping on the structural and electronic properties of NC supports and Pd/NC catalysts were investigated by XRD, N_2_ physisorption, XPS, H_2_-O_2_ titration and TEM. The relationship between nitrogen doping and its impacts on hydrogenation performance of Pd/NC catalysts are discussed in detail.

## Experimental section

### Materials

All chemical agents were commercially available. They were used without further purification. The reactant 4-CBA were obtained from Aladdin Chemical Reagent (Shanghai, China). Palladium chloride was purchased from Sigma-Aldrich. Activated carbon was provided by Sinopec Shanghai Petrochemical Company Limited. Zinc nitrate hexahydrate, 2-methylimidazole, phosphoric acid (H_3_PO_4_, 85 wt% in H_2_O), ammonia solution (NH_3_ ▪ H_2_O, 25 wt% in H_2_O) were purchased from Sinopharm Chemical Reagent Co., Ltd.

### Synthesis of NC samples

Firstly, ZIF-8 was supported on AC according to the previous report with few modifications (Li J. et al., 2016). Typically, the activated carbon (C) was dispersed in the solution of 0.6 g Zn(NO_3_)_2_·6H_2_O with 8 ml ethanol and 2 ml water. 1.31 g 2-methylimidazole dissolved in the mixture of 8 ml ethanol and 2 ml water. Then the solution of 2-methylimidazole was added into the above salt solution with stirring for 20 min. The mixed solution was transferred into a 100 ml Teflon-lined stainless autoclave. After sealed, the autoclave was heated to 120°C and maintained for 2 h. The hydrothermal product was filtered and washed thoroughly by water, and finally dried at 110°C. The above obtained samples (C@ZIF-8) were carbonized at different temperatures (500°C, 600°C, 700°C, and 800°C) for 2 h in nitrogen atmosphere. The carbonized samples were treated by HCl aqueous solution and washed by water. After drying at 120°C, the final samples were denoted as NC-T, where T represented the calcination temperature of 500°C, 600°C, 700°C, and 800°C.

## Preparation of Pd/NC catalysts

The Pd catalysts was prepared by an impregnation method using aqueous solution of PdCl_2_. The theoretical loading contents of Pd on NC samples were 0.5 wt%. The Pd catalysts were denoted as Pd/NC-T, in which T represented 500°C, 600°C, 700°C, and 800 C. For comparison, activated carbon without nitrogen doping supported Pd catalyst (Pd/C) was also prepared with the same method as described above.

### Materials characterization

Powder X-ray diffraction (XRD) measurement was carried out using Bruker D8 Advance diffractometer, with Cu Kα radiation (g = 0.15406 nm) at step scan 0.02° from 5° to 80°. The nitrogen adsorption/desorption isotherms were obtained at 77 K on a Micromeritics ASAP 2020 analyzer. The total porous volume (V_total_) was estimated from the adsorbed capacity of nitrogen at a relative pressure P/P_0_ of 0.98. The microporous volume (V_micro_) was determined using the t-plot method. X-ray photoelectron spectroscopy (XPS) analysis was performed with an AXIS Ultra DLD photoelectron spectrometer (Kratos Analytical, United Kingdom) using monochromatic Al Kα radiation. The C1s peak at 284.6 eV was applied as reference to calibrate the position of the other peaks. Palladium dispersion on NC samples were tested by hydrogen titration of chemisorbed oxygen (H_2_-O_2_ titration) in a Micromeritics Autochem II 2920 instrument with a thermal conductivity detector (TCD). Before the tests, the Pd catalysts were firstly reduced under a hydrogen atmosphere at 200°C for 60 min. Typically, 400 mg catalyst sample was pre-reduced at 200°C by hydrogen for 120 min. After pre-reduction, hydrogen absorbed on the surface of catalyst was titrated by 15 pulses of oxygen until full saturation. Furthermore, the resulting monolayer of adsorbed oxygen was titrated using 15 pulses of hydrogen until constant TCD signal detected. The content of Pd loading was tested by an inductively coupled plasma emission spectrometer (ICP-OES, Varian 725ES). The hydrogen temperature programmed desorption (H_2_-TPD) experiments was carried out using a Micromeritics Autochem II 2920 instrument equipped with a thermal conductivity detector (TCD). HRTEM images and elemental mapping of Pd catalysts were conducted on a Tecnai 20 S-TWIN electron microscope operated at 200 kV.

### Catalytic hydrogenation tests

The Hydrogenation of 4-CBA was performed in a 100 ml stainless autoclave with a Teflon liner. Generally, 50 mg catalyst and 0.15 g 4-CBA were dispersed in 50 ml de-ionized water. After the autoclave was sealed, it was purged by hydrogen to remove the air. Then it was pressured with 0.8 MPa hydrogen. Finally, the autoclave was heated to 80°C for hydrogenation reaction. After the completion of this reaction, the obtained liquid products were treated with 14% NH_3_·H_2_O and 17% H_3_PO_4_ aqueous solutions respectively. Finally, the liquid products were analyzed through high-performance liquid chromatography (HPLC, Agilent 1,200). The HPLC was equipped with a UV detector and a C-18 column (4.6*250 mm, 5 μm). The hydrogenation of 4-CBA over Pd catalysts leaded to primary product 4-hydroxymethyl benzoic acid. The TOF (turnover frequency) of Pd catalysts was calculated as the number of moles of 4-CBA converted per mole of Pd per minute.

## Results and Discussion

### Effects of nitrogen doping on the structure of NC supports

**FIGURE 1:**
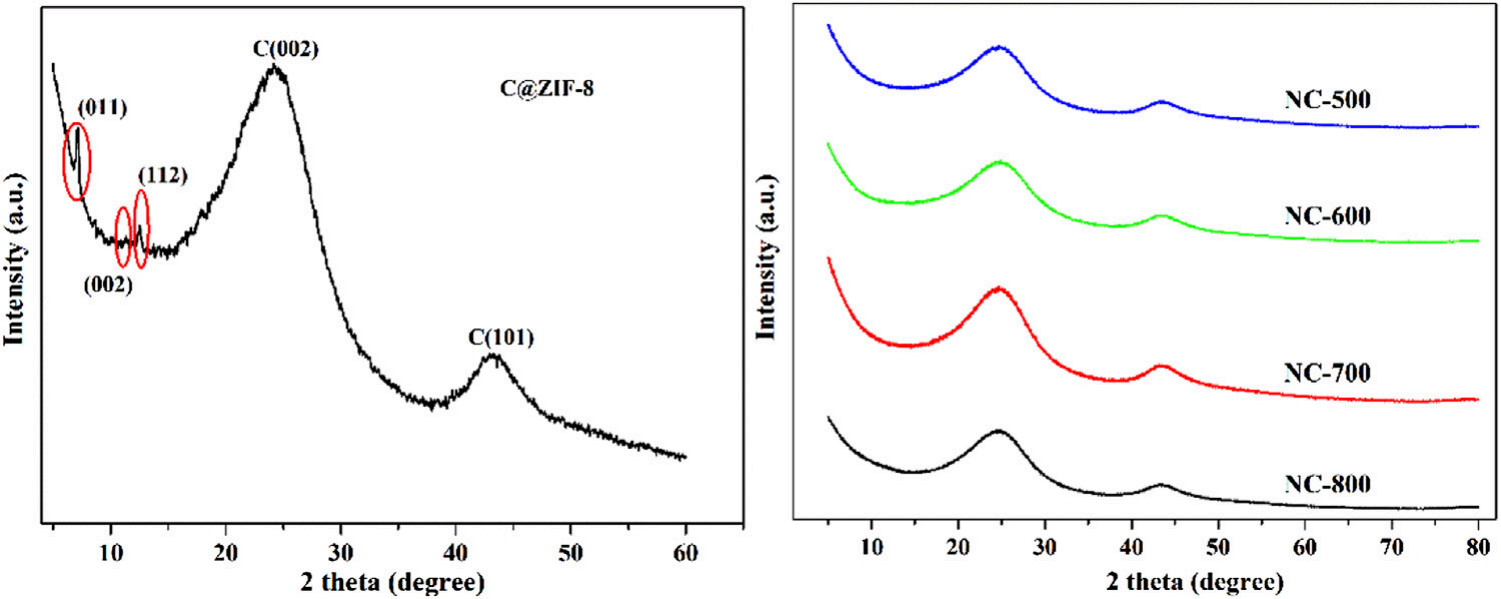
XRD spectra of ZIF-8-C-pre and NC samples.

All of NC samples in present work were synthesized in similar procedure but at different calcination temperatures ranging from 500 to 800°C to tailor different N species. Firstly, XRD patterns of C@ZIF-8 and NC samples were shown in Figure 1. Before calciantion, the precursor C@ZIF-8 showed the diffraction of ZIF-8 (cubic space group), corresponding to the ZIF-8 (011) (002) and 112) diffractions, respectively (Zhang et al., 2014). After calcination at different temperature, four NC samples exhibited similar diffraction peaks at around 25° and 43°, corresponding to the graphitic carbon (002) and (100) diffraction peaks (Fu et al., 2017). Further, nitrogen adsorption-desorption isotherms of NC samples were tested and the structure parameters of these materials were summarized in Table 1. NC-500 exhibited the lowest BET surface area (678.1 m^2^/g). NC-600 showed higher BET surface area (701.6 m^2^/g) than that of NC-500. NC-700 and NC-800 have similar BET surface area with NC-600. These results demonstrated that nitrogen species of NC can be regulated by different pyrolysis temperature at 600–800°C, while similar surface area and porous volume of NC were preserved.

**TABLE 1:** Summary of porosity parameters of NC samples.

Sample	S_BET_ (m^2^/g)	S_mic_ m^2^/g	V_t_ (cm^3^/g)	V_micro_ (m^3^/g)
NC-500	678.1	548.9	0.33	0.25
NC-600	701.6	552.2	0.34	0.25
NC-700	707.3	582.1	0.34	0.26
NC-800	704.1	614.1	0.32	0.28

The specific surface areas (S_BET_) were calculatd by the Brunauer-Emmett-Teller (BET) method. V_t_ denoted the total pore volume. The micropore area (S_mic_) and micropore volume (V_micro_) was determined by t-plot method.

**FIGURE 2:**
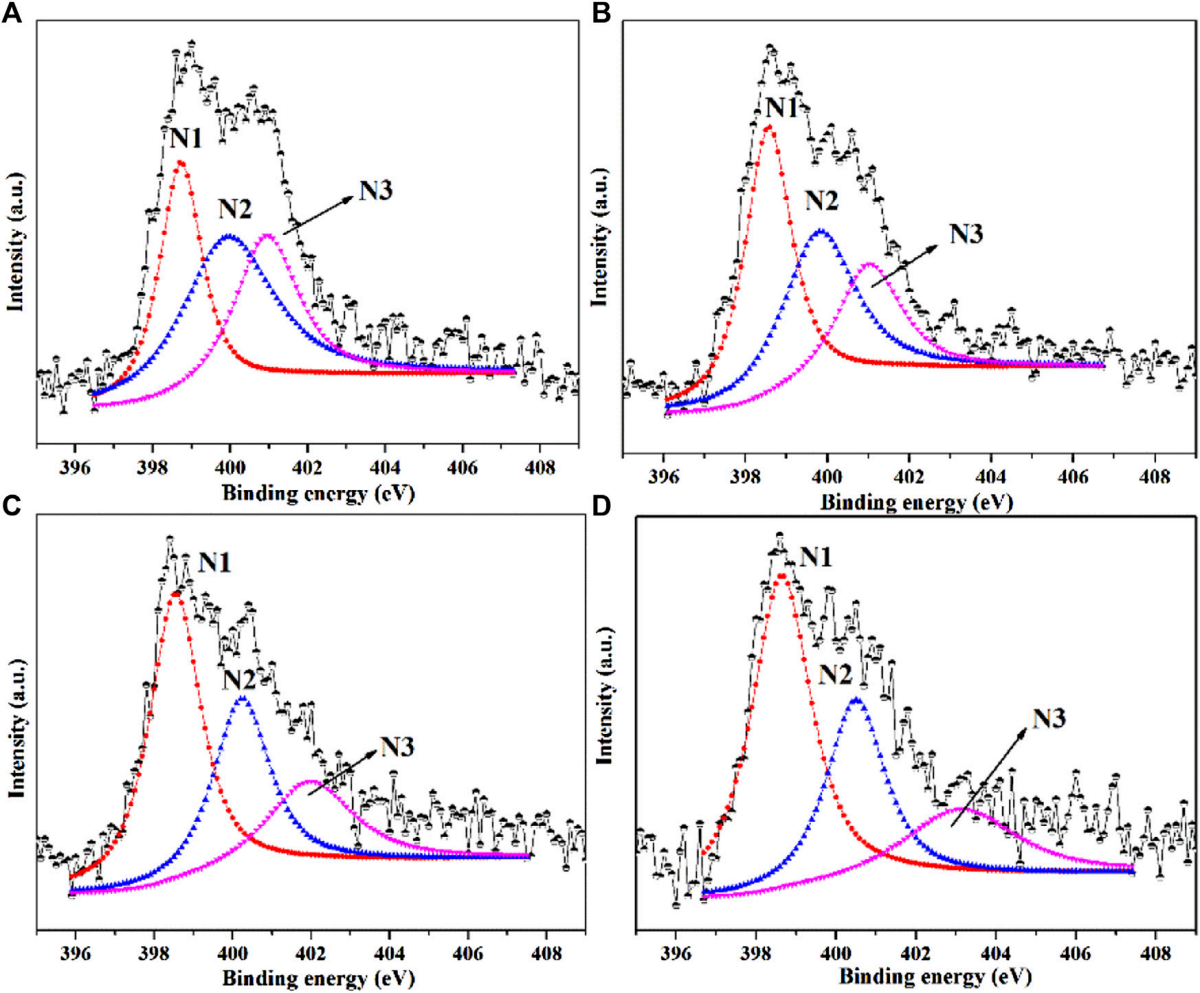
N 1s XPS spectra of **(A)** NC-500, **(B)** NC-600, **(C)** NC-700 and **(D)** NC-800. (N1: pyridinic N; N2: pyrrolic N; N3: graphitic N).

**SCHEME 1:**
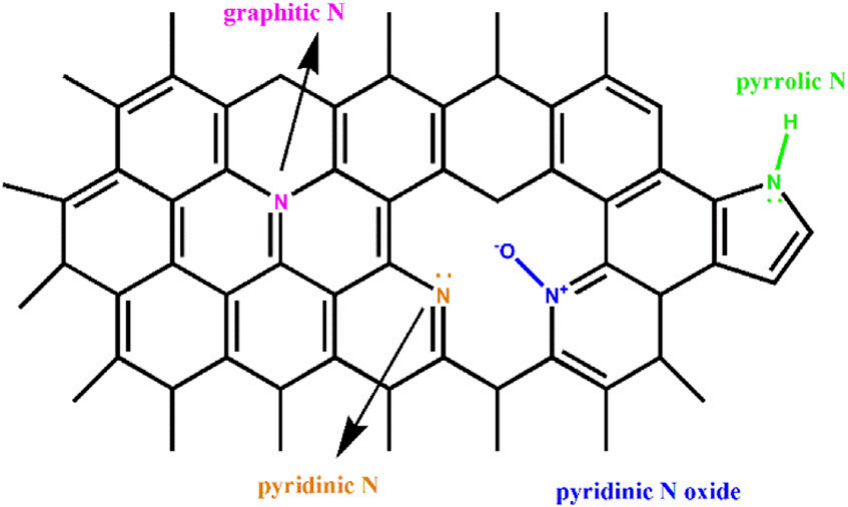
Nitrogen types in nitrogen doped carbon materials.

**FIGURE 3:**
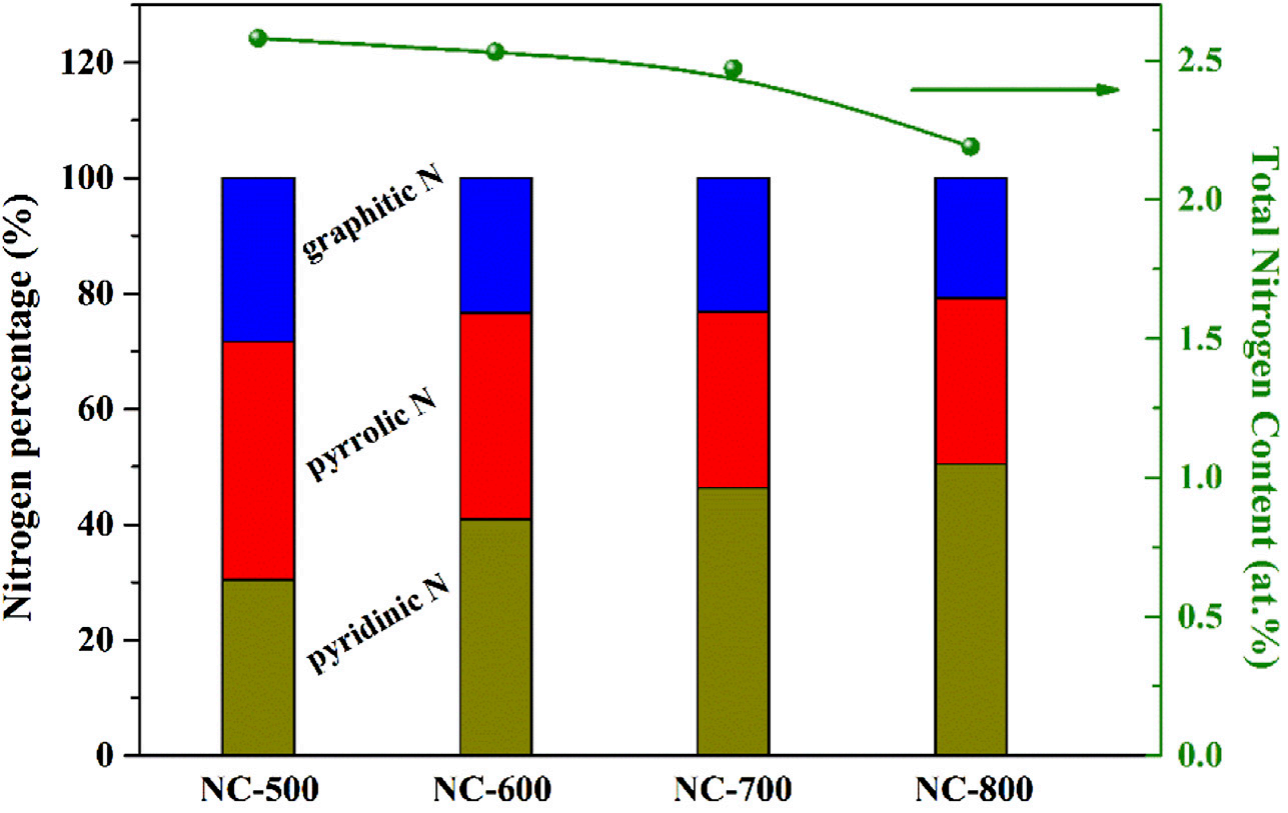
N contents and N types of different NC samples.

XPS characterization was performed to analyze N types and N contents of NC samples (Peng et al., 2014; Fu et al., 2017; Yang et al., 2018). As shown in Figure 2, the high-resolution N1s XPS spectra of four NC samples revealed there were mainly three types of N species: pyridinic N (N1) at 398.5 eV, pyrrolic N (N2) at 399.8 eV and graphitic N at 401.9 eV (N3) (Xiong et al., 2014; Chen et al., 2015). The typical nitrogen types in nitrogen doped carbon materials were shown in Scheme 1 (Li M. et al., 2016; Cao et al., 2017; Quílez-Bermejo et al., 2019). In Figure 3, the total N content decreased from 2.58 to 2.19 at.% with the increasing of calcination temperature. The relative contents of different N species in Figure 3 were calculate based on the integration areas of each N1s peak component from the N1s XPS spectra in Figure 2. The relative percentage of pyridinic N increased from 30.5% to 50.5%. Differently, the percentage of pyrrolic N decreased continuously with increasing calcination temperature. Similar results have been reported earlier for N doping carbon materials, which revealed that pyrrol functional groups were not stable at high temperature and partly transformed to pyridinic N (Arrigo et al., 2008; Sun et al., 2017).

## Effects of nitrogen doping on the electronic structure of Pd/NC catalysts

**FIGURE 4:**
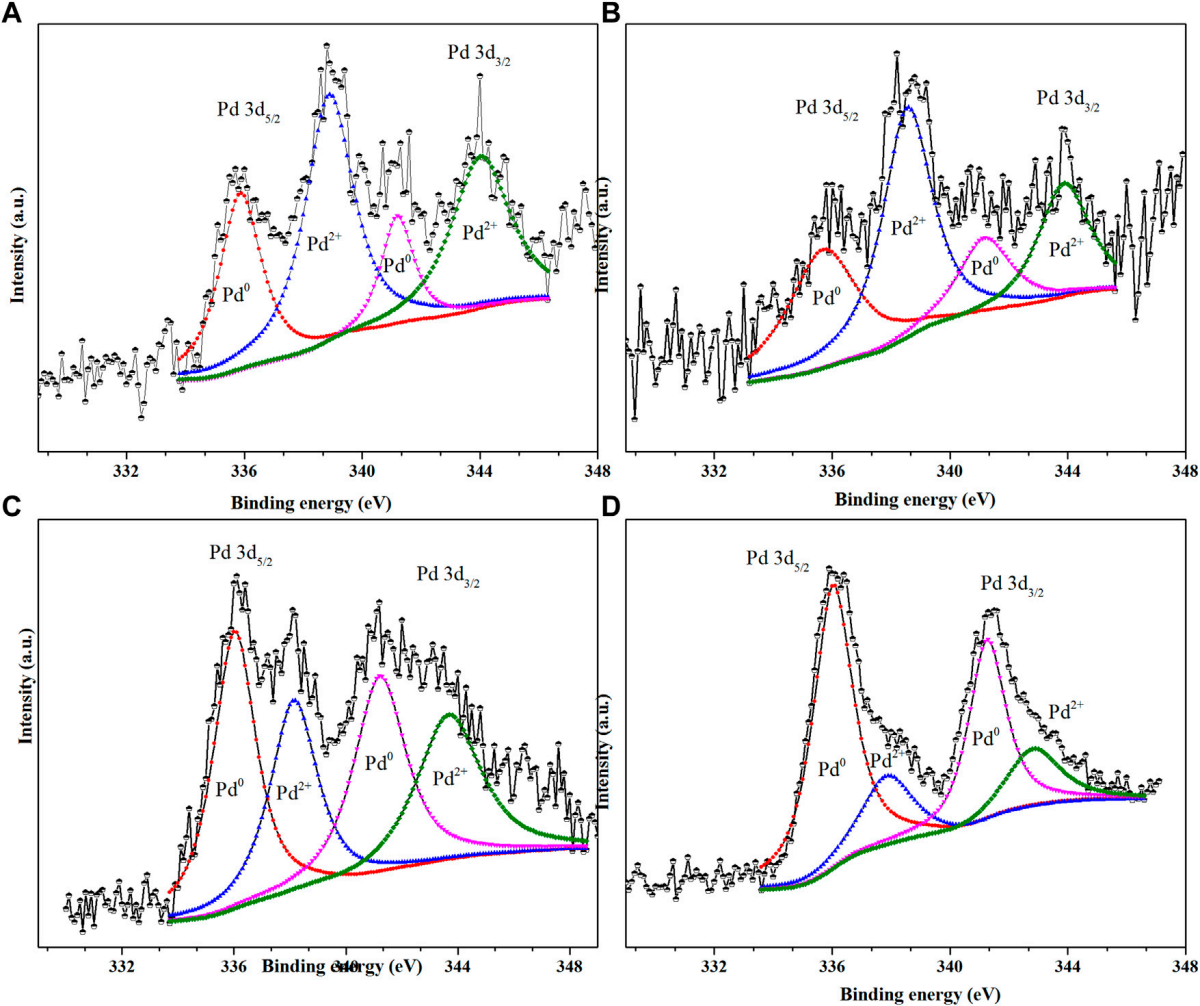
Pd 3d XPS spectra of **(A)** Pd/NC-500, **(B)** Pd/NC-600, **(C)** Pd/NC-700 and **(D)** Pd/NC-800.

**FIGURE 5:**
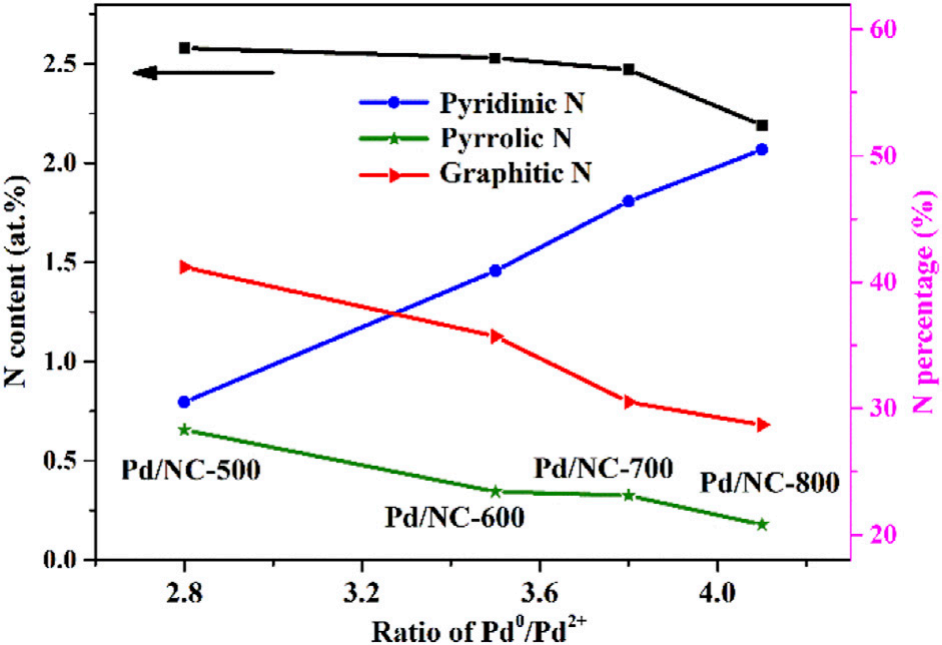
Relation of N content and N types with the ratio of Pd^0^/Pd^2+^.

The influence of N doping on the electronic structures of Pd NPs was also studied by XPS. As shown in Figure 4, the four Pd 3d XPS spectra was fitted using four separated components with peaks at 335.8, 341.2, 337.3, and 342.5 eV. The former two peaks were ascribed to Pd^0^ species (Pd^0^ 3d_5/2_, Pd^0^ 3d_3/2_). The latter two peaks were assignable to Pd^2+^ species (Pd^2+^ 3d_5/2_, Pd^2+^ 3d_3/2_) (Xu et al., 2012). As illustrated in Figure 5, the ratio of Pd^2+^ against Pd^0^ (Pd^0^/Pd^2+^) gradually increased with the temperature of calcination from 500°C to 800°C. As revealed in Figure 5, the ratio of Pd^0^/Pd^2+^ had only weak dependence on N content. However, the ratio of Pd^0^/Pd^2+^ showed strong dependence on pyridinic N. This indicated that pyridinic N as preferential anchoring sites could favor the formation of high ratio of Pd^0^/Pd^2+^ by donating electrons toward neighboring Pd nanoparticles.

Besides, Pd dispersion (D_Pd_) was evidenced by H_2_-O_2_ titration analysis. As shown in Table 2, Pd/C showed the lowest Pd dispersion. However, NC as the supports of Pd nanoparticles exhibited well dispersed with D_Pd_ higher than 25%. Previous studies indicated that carbon materials with N doping favored well dispersion of metal nanoparticles compared to the un-doped ones (Shen et al., 2019; Hu et al., 2022). For example, Warczinski et al. has confirmed that loading of Pd on N-doped mesoporous carbon leaded to a better dispersion of Pd compared to those for Pd supported on N-free mesoporous carbon (Warczinski et al., 2020). In present work, N functional groups on the NC supports played a crucial role in effectively preventing the aggregation of PdNPs and anchoring the formed PdNPs.

**TABLE 2:** Physico-chemical properties of Pd catalysts.

Catalyst	Pd loading (wt%)	D_Pd_ (%)
Pd/C	0.40	21.3
Pd/NC-500	0.42	25.1
Pd/NC-600	0.41	25.3
Pd/NC-700	0.41	27.3
Pd/NC-800	0.42	32.6

## Effects of nitrogen doping on the activity of Pd/NC

**FIGURE 6:**
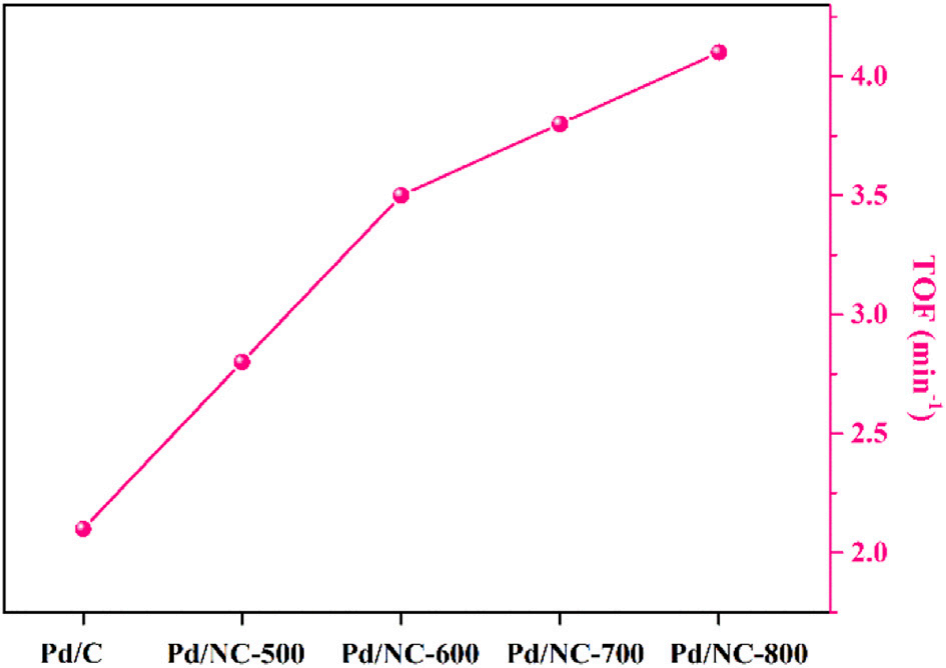
Catalytic performances of Pd catalysts for the hydrogenation of 4-CBA.

In order to elucidate the effect of N doping, the catalytic performances of Pd catalysts with and without N doping were investigated for the hydrogenation reaction of 4-CBA. As shown in Figure 6, activated carbon supported Pd catalyst (Pd/C) showed the lowest catalytic activity. The activity of Pd/NC-500 was higher than that of Pd/C. The activity of Pd/NC-600 and Pd/NC-700 further increased. The Pd/NC-800 showed highest TOF value of 4.1 min^−1^. These catalytic results indicated TOF values (Figure 6) for the N-doped Pd catalysts were higher than Pd/C catalyst. The important promotion effects of N doping were further discussed as below.

### Structure and activity relationship

The change of the ratio of Pd^0^/Pd^2+^ was related with the different N content and N types of supports NC (Figure 5). As mentioned in Figure 3, the N content of NC decreased along with the increase of calcination temperature from 500°C to 800 C. However, the catalytic activities of Pd/NC increased with the increasing of calcination temperature (Figure 6). The variation trend of TOF value of Pd/NC did not agree with the effect of N content for 4-CBA hydrogenation. Thus, the N content was not the primary factor to influence hydrogenation activity, but the moderate N content may be a positive effect on this hydrogenation reaction. It was noticed that the ratio of Pd^0^/Pd^2+^ in Pd/NC catalysts increased almost linearly along with the relative percentage of pyridinic N (Figure 5). The highest ratio of Pd^0^/Pd^2+^ showed the best hydrogenation performance. Owing to its electron donating properties, pyridinic N can enhance the interaction between Pd and NC through its lone pairs as metal coordination sites, preventing the reoxidation of Pd^0^. These results were in line with some experimental and DFT studies of pyridinic nitrogen strong interaction with metal nanoparticles (Li et al., 2016c; Nagpure et al., 2016; Jia et al., 2021). Besides, H_2_-O_2_ titration results indicated that Pd nanoparticles loading on NC supports maintained better dispersion compared with that of un-doped one.

**FIGURE 7:**
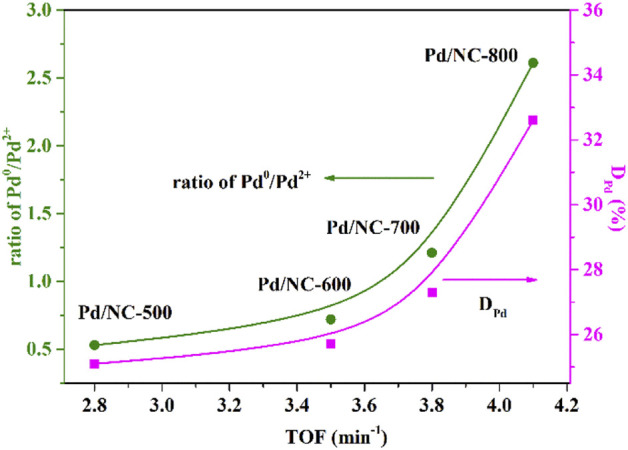
Dependence of D_Pd_ and Pd^0^/Pd^2+^ on catalytic performance of Pd/NC.

To further clarify the important roles of nitrogen doping on catalytic activity of Pd/NC catalysts, the relationships of TOF of four Pd/NC catalysts and the ratio of Pd^0^/Pd^2+^ as well as Pd dispersion were depicted in Figure 7. A strong dependence of the TOF of Pd/NC catalysts with the ratio of Pd^0^/Pd^2+^ as well as Pd dispersion was observed. The higher the ratio of Pd^0^/Pd^2+^, the higher the catalytic activity of Pd/NC catalysts. Besides, the higher Pd dispersion also resulted in higher activity for Pd/NC catalysts. The above linear relationship may be attributed to the more exposure active sites Pd^0^ over Pd/NC.

**FIGURE 8:**
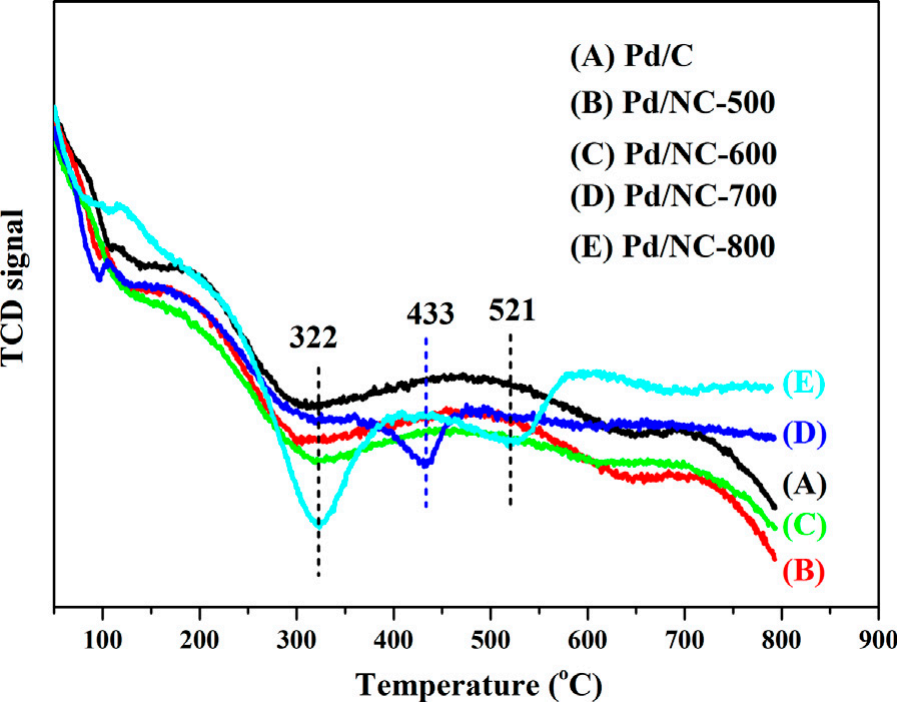
H_2_-TPD spectra of **(A)** Pd/C, **(B)** Pd/NC-500, **(C)** Pd/NC-600, **(D)** Pd/NC-700 and **(E)** Pd/NC-800.

**FIGURE 9:**
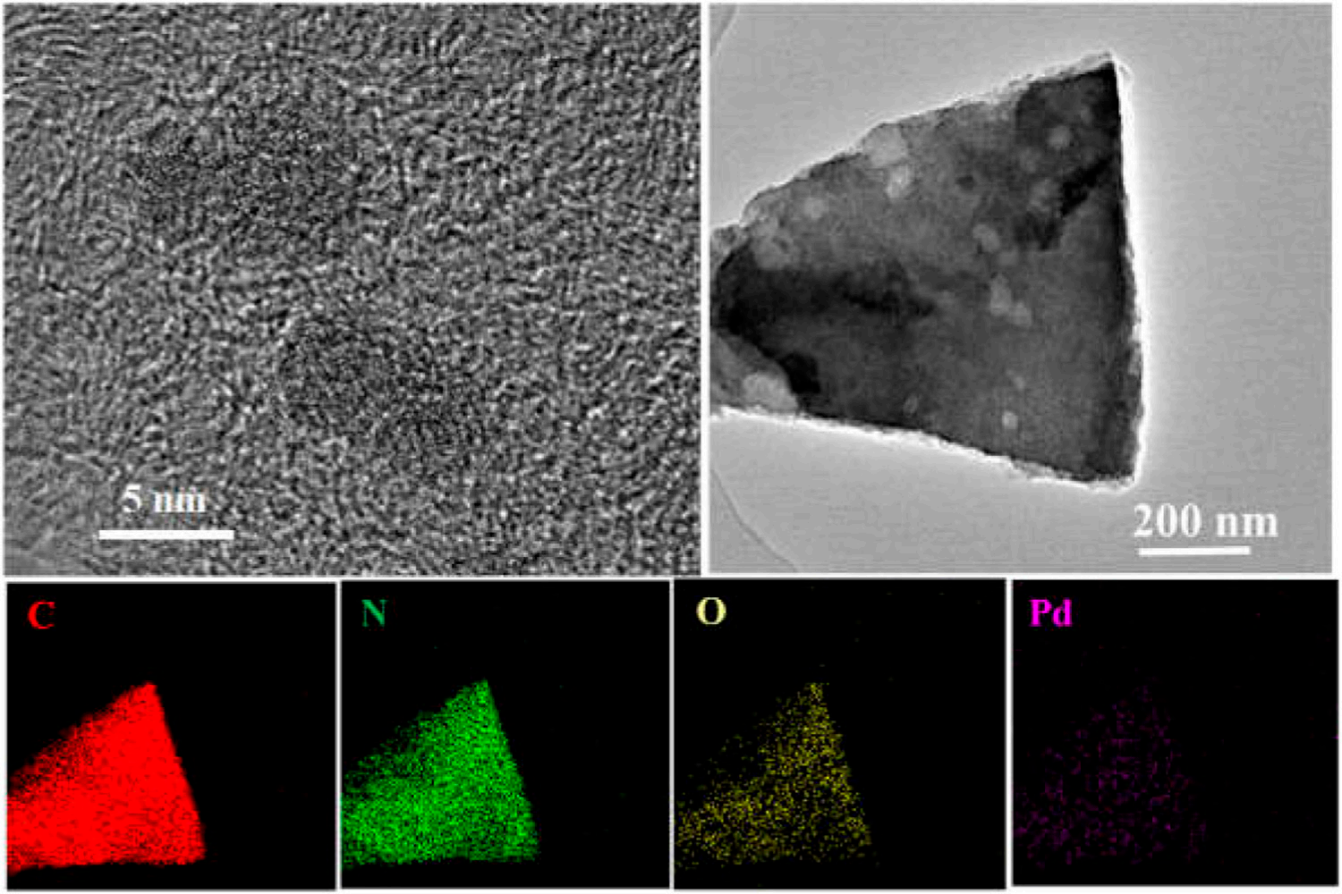
Representative HRTEM images of Pd/NC-800 and the EDS elemental mapping of C, N, O and Pd.

**FIGURE 10:**
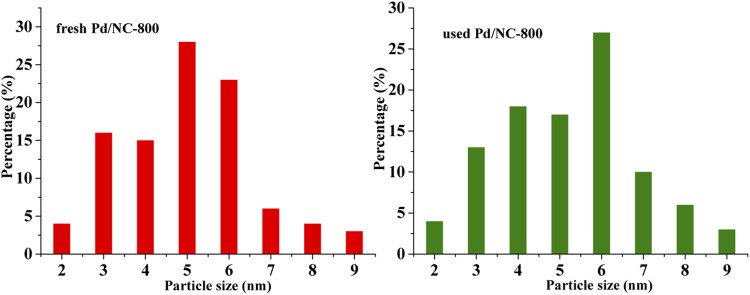
Particle size distributions of Pd NPs in fresh and used Pd/NC-800.

It was well known that the hydrogen dissociation/activation ability has a profound effect on catalytic hydrogenation performance (Liu et al., 2016; Bi et al., 2019). The H_2_ activation/dissociation ability of Pd catalysts were studied *via* H_2_-TPD. As shown in Figure 8, all catalysts showed a similar hydrogen desorption peak at 322°C, indicating that similar type of active sites existed on Pd catalysts surface. In addition, there was an obvious desorption peak at 433°C for Pd/NC-700. Pd/NC-800 showed two desorption peaks at low temperature of 322°C and high temperature of 521°C. The differences of desorption peaks indicated there were different types of active Pd sites on Pd/NC-700 and Pd/NC-800. The desorption peak at low 322°C was assigned to chemisorbed hydrogen on surface Pd active sites, and the high temperature peaks at 433 and 521°C were assigned to strongly chemisorbed hydrogen on different types of Pd active sites. Furthermore, the total hydrogen desorption of Pd/NC-800 were obviously larger than that of other Pd catalysts. Therefore, H_2_-TPD results further concluded that the enhanced activation capability of H_2_ molecular in NPC-800 supported Pd catalyst also contributed to its higher hydrogenation activity. Furthermore, the morphologies of Pd NPs and the EDS elemental mapping of C, N, O and Pd of Pd/NC-800 was shown in Figure 9. It was evident that N and Pd were dispersed uniformly on the surface of Pd/NC-800. In addition, the used Pd/NC-800 catalyst was centrifuged and washed with ethanol for further TEM analysis. The particle size distributions of Pd in fresh and used Pd/NC-800 were shown in Figure 10. These results indicated that Pd nanoparticles in used Pd/NC-800 still maintained well dispersion and remained similar particle size distribution compared with that of fresh Pd/NC-800. In addition, Pd content in used Pd/NC-800 was also analyzed by ICP-OES technique. The results confirmed that the leaching of Pd was negligible. These results indicated Pd/NC-800 catalyst was quite stable. Therefore, the present NC materials can be promising catalyst supports to immobilize Pd nanoparticles (Li et al., 2013; Yu et al., 2021).

## Conclusion

In summary, the present work demonstrated the comprehensive understanding of N doping on the catalytic activities of NC supported Pd catalysts for the catalytic hydrogenation of 4-CBA. The Pd/NC catalysts exhibited enhanced hydrogenation activity compared with Pd/C. Pd/NC-800 with 50.5% of pyridinic nitrogen exhibited excellent activity of 4.1 mim^−1^. The excellent catalytic activity of Pd/NC-800 could ascribed to a high density of active species Pd^0^ where the Pd^0^ were confirmed to be mostly coordinated with pyridinic nitrogen. Furthermore, nitrogen species can also improve the dispersion of Pd NPs *via* strong interaction between Pd and pyridinic nitrogen. The Pd dispersion of Pd/NC-800 was up to 32.6%. Besides, the well dispersion of Pd nanoparticles can maintain even after reaction. Thus, pyridinic nitrogen species coordinated with active species Pd^0^ also contributed to the high stability of Pd/NC catalysts. More importantly, the results of H_2_-TPD tests clearly demonstrated that nitrogen species promoted the adsorption and activation capability of H_2_ molecular. Particularly, H_2_-TPD results also confirmed that new Pd active sites were created on the surface of Pd/NC catalysts. Therefore, the as-synthesized NC materials may be the promising supports of other supported metal catalysts, which are helpful for the dispersion and stabilization of metal nanoparticles.

## Data Availability

The original contributions presented in the study are included in the article/Supplementary Material, further inquiries can be directed to the corresponding authors.
